# Supplementary material for: Association between oral microbiome and seven types of cancers in East Asian population: a two-sample Mendelian randomization analysis
Source: Front Mol Biosci. 2023 Nov 21;10:1327893. doi: 10.3389/fmolb.2023.1327893 (PMC10702768; doi:10.3389/fmolb.2023.1327893)
Supplement: Supplementary file 1 [file DataSheet1.ZIP › Supplementary Materials/MR plots of saliva/saliva═╝/Breast cancer/pheno.895_to_breast cancer_leave_one_out.pdf]

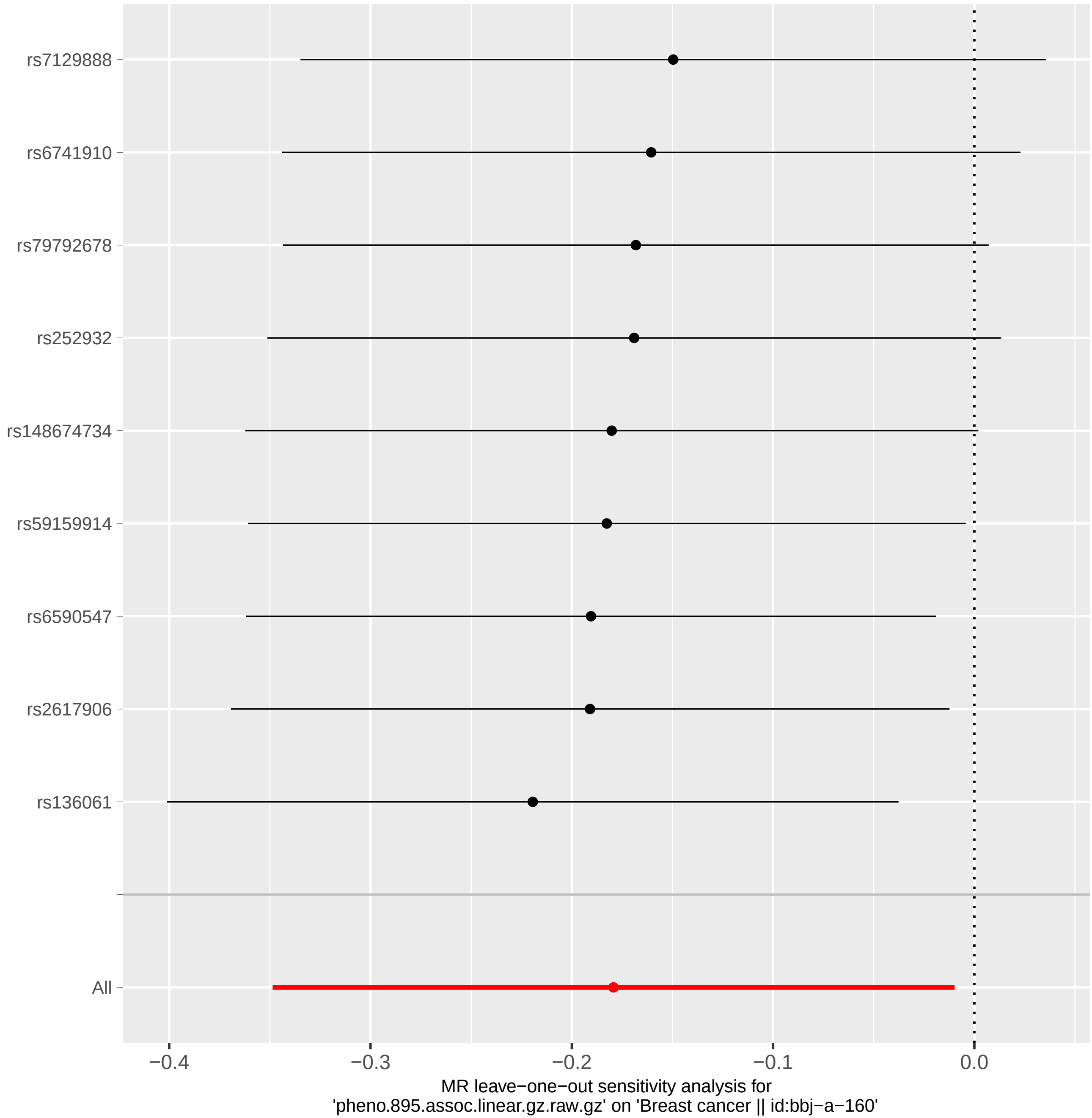
rs7129888
rs6741910
rs79792678
rs252932
rs148674734
rs59159914
rs6590547
rs2617906
rs136061
All
−0.4
−0.3
−0.2
−0.1
0.0
MR leave−one−out sensitivity analysis for
'pheno.895.assoc.linear.gz.raw.gz' on 'Breast cancer || id:bbj−a−160'
